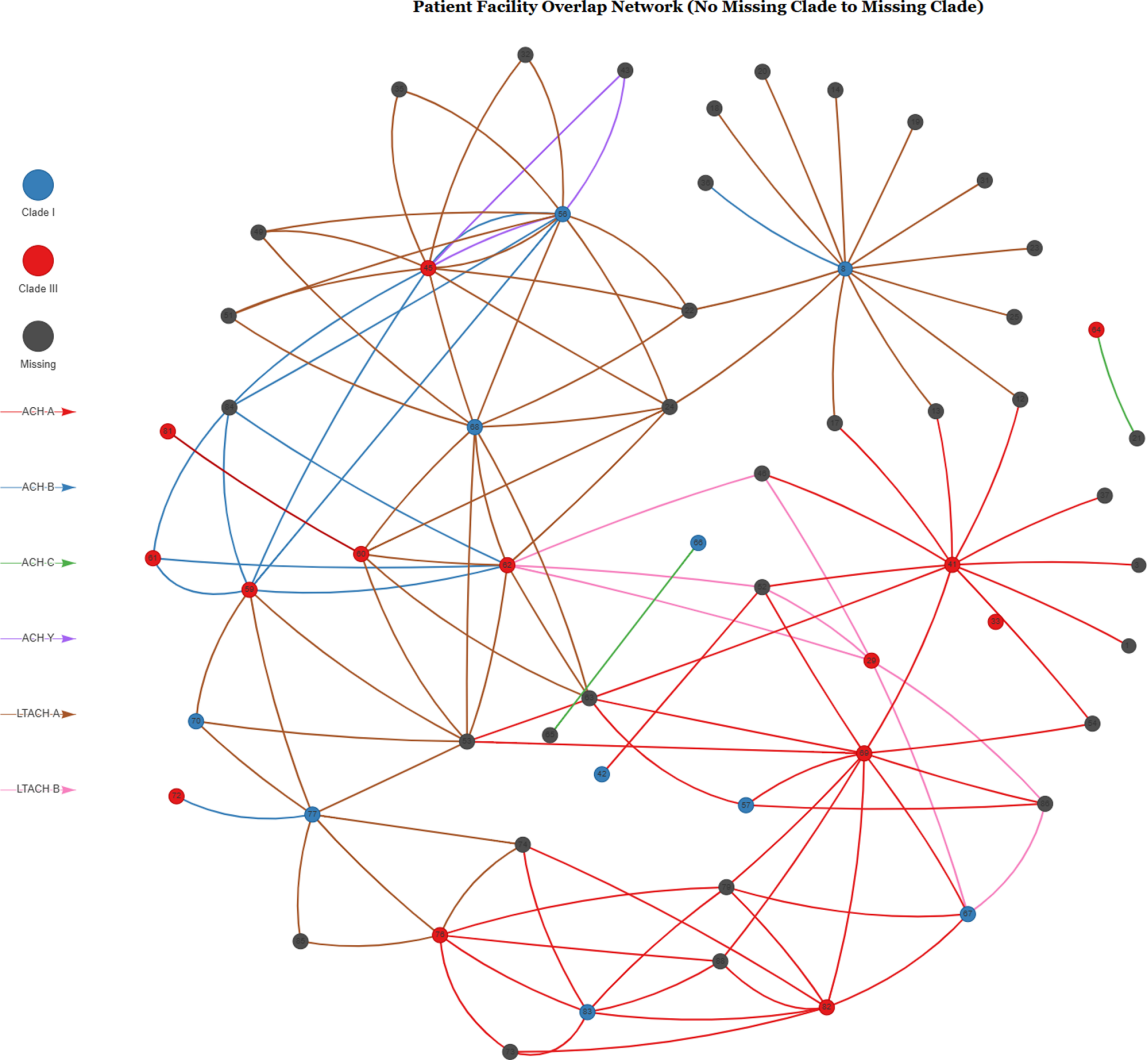# 44 Facility-level variation in antibiotic prescribing for outpatient low-risk diverticulitis in the Veterans Health Administration

**DOI:** 10.1017/ash.2026.10482

**Published:** 2026-06-23

**Authors:** Michael Anderson, Benjamin Roberts, Dhondup Urg, Diane Yi, Shamta Warang, Lynn Reynolds, Sue Milligan, Karen Williams, JoAnna Wagner

**Affiliations:** 1 Georgia Department of Public Health

## Abstract

**Background:** Candidozyma auris is an emerging multidrug-resistant organism often associated with multi-facility outbreaks. We identified an outbreak in southeast Georgia spanning September 2024-August 2025. Although early response efforts focused on one long-term acute care hospital (LTACH) with extensive colonization, clinical cases were also identified in four acute care hospitals (ACHs), another LTACH, and one outpatient center within the public health district. Two ACH laboratories were only speciating Candida from blood cultures. We expanded the investigation to assess whether inter- and intra-facility transmission contributed to ongoing spread, to inform targeted testing, colonization screening, and infection control recommendations. **Method:** Colonization was detected in LTACH-A-LTACH-B via Polymerase Chain Reaction by Tennessee Laboratory Services, and clinical cases were identified via MALDI-TOF spectrometry at the Georgia Public Health Laboratory or clinical laboratories. Whole-genome sequencing (WGS) was conducted on 24 isolates. Clade data were analyzed alongside patient healthcare exposure histories to identify transmission clusters. Case data were managed in Excel, and healthcare exposure networks were visualized using visNetwork in RStudio. **Result:** C. auris cases were identified across 7 facilities, including ACH-A-ACH-D, LTACH-A-LTACH-B, and Outpatient-A. We identified 26 clinical and 62 screening cases. Clinical specimen sources included 31% blood, 27% wound, 23% urine, 15% respiratory, and 4% unknown. LTACH-A identified 3 clinical cases in Oct./Nov. 2024, leading to screening identifying 60 cases between 12/3/24-8/5/25. Of 60 screening cases in LTACH-A, 37% had prior exposure to ACH-A, 25% ACH-B, 17% ACH-C, and 2% ACH-D. 17 clinical cases were identified in ACH-A-ACH-D between 9/4/24-7/23/25; no screening was conducted. Review of patient healthcare exposure history uncovered 12 overlap clusters of 2-6 cases: 8 ACH-A (multiple high-acuity unit overlaps), 3 ACH-B (ICU & Med/Surg), and 1 ACH-C (no shared unit). Clinical cases in each cluster overlapped with ≥1 cases that later screened positive in LTACH-A. WGS results showed 10 (42%) clade-I and 14 (58%) clade-III. 3 ACH clusters were identified with clade-III cases, 4 had clade-I and clade-III, and 7 lacked WGS results. **Conclusion:** Integration of healthcare network analysis with limited WGS data demonstrated that C. auris transmission occurred within ACHs and across the public health district, rather than confined to one LTACH. Patient overlap clusters and temporal sequencing of clinical ACH cases preceding LTACH-A colonization results supported ACH-driven transmission contributing to downstream spread. These findings informed targeted recommendations for speciating non-blood cultures, implementing admission screening at ACHs, and increasing WGS capacity to strengthen multi-facility outbreak detection and response.

**Figure:**